# Effect of bone material properties on effective region in screw-bone model: an experimental and finite element study

**DOI:** 10.1186/1475-925X-13-83

**Published:** 2014-06-21

**Authors:** Shuai Liu, Wei Qi, Yang Zhang, Zi-Xiang Wu, Ya-Bo Yan, Wei Lei

**Affiliations:** 1 Department of Orthopaedics, Xijing Hospital, Fourth Military Medical University, Xi’an 710032, Shaanxi Province, P.R. China; 2 Surgery Department of 520th Hospital of PLA, Mian yang, China

**Keywords:** Biomechanics, Pedicle screw, Cancellous bone, Finite element analysis

## Abstract

**Background:**

There have been numerous studies conducted to investigate the pullout force of pedicle screws in bone with different material properties. However, fewer studies have investigated the region of effect (RoE), stress distribution and contour pattern of the cancellous bone surrounding the pedicle screw.

**Methods:**

Screw pullout experiments were performed from two different foams and the corresponding reaction force was documented for the validation of a computational pedicle screw-foam model based on finite element (FE) methods. After validation, pullout simulations were performed on screw-bone models, with different bone material properties to model three different age groups (<50, 50–75 and >75 years old). At maximum pullout force, the stress distribution and average magnitude of Von Mises stress were documented in the cancellous bone along the distance beyond the outer perimeter pedicle screw. The radius and volume of the RoE were predicted based on the stress distribution.

**Results:**

The screw pullout strengths and the load–displacement curves were comparable between the numerical simulation and experimental tests. The stress distribution of the simulated screw-bone vertebral unit showed that the radius and volume of the RoE varied with the bone material properties. The radii were 4.73 mm, 5.06 mm and 5.4 mm for bone properties of ages >75, 75 > ages >50 and ages <50 years old, respectively, and the corresponding volumes of the RoE were 6.67 mm^3^, 7.35 mm^3^ and 8.07 mm^3^, respectively.

**Conclusions:**

This study demonstrated that there existed a circular effective region surrounding the pedicle screw for stabilization and that this region was sensitive to the bone material characteristics of cancellous bone. The proper amount of injection cement for augmentation could be estimated based on the RoE in the treatment of osteoporosis patients to avoid leakage in spine surgery.

## Background

Osteoporosis is a common skeletal disorder of the spine and hip in the elderly population. Spine surgeons often encounter patients with osteoporotic spines that require spinal decompression and management with surgical instrumentation due to degenerative and traumatic spinal diseases [1,2]. Pedicle screw fixation is a routine tool for spine stabilization, with the screw providing rigid bony secured points on internal fixation devices. However, it is a challenge for spine surgeons to perform pedicle screw instrumentation surgery on osteoporotic spines to prevent many potential complications, such as screw loosening, migration or back-out [3]. It has been reported that the mechanical strength of the bone-screw interface is adversely affected by low bone density in patients with osteoporosis [4,5].

Different methods have been used to enhance the short- and long-term stability of implanted screws in the osteoporotic lumbar spine [6,7]. The in situ injection of biomaterials, such as calcium phosphate cement (CPC), calcium sulfate cement (CSC), and polymethyl-methacrylate (PMMA), into the screw hole is a common option for enhancing pedicle screw fixation strength [8,9]. Although pedicle screw augmentation with cement is an attractive option for improving screw fixation, there exist risks of excessive cement leakage beyond the confined target bone, affecting the spinal cord and resulting in nerve compression [10]. A smaller-volume cement injection might not enhance the augmentation screw performance.

Although there have been many experimental screw-bone interaction studies, it is technically difficult to determine the region of effect (RoE) by observing the screw-bone interaction during pullout testing. Sources in the literature have reported that the pullout strength of the pedicle screw increased from 147% to 300% [9-12] when the amount of cement injection varied from approximately 1 to 3.5 ml. Liu et al. [7] and Chang et al. [9] demonstrated that an appropriate volume of injection could be obtained by investigating of the interaction between the pedicle screw and cancellous bone. Compared to experimental models, finite element (FE) models provide the opportunity to document related mechanical responses during simulation [13]. Some researchers have used FE models for screw-bone interaction studies. Moazen M et al. [14] evaluated the screw–bone interface model in a locking plate fixation through a corroboration study. Zhang et al. [15] developed a quarter screw-bone model and studied the effects of the bone material on the screw pullout strength. Chatzistergos et al. [16] used a two-dimensional screw-bone model to perform a parametric study of pedicle screw design. Because the pullout force is set as the dominant index for the evaluation of screw fixation, there have been numerous studies conducted regarding in this aspect, with fewer researchers investigating RoE, stress distribution and the contour pattern of the cancellous bone surrounding the pedicle screw.

Based on the pullout experiments [6,7,17] and micro-structural studies of the vertebral cancellous bone [18,19], we speculated that there existed an effective region or an enclosed RoE around the perimeter of the pedicle screw that might play a pivotal role in the stabilization of the pedicle screw during screw pullout. Therefore, the purpose of this study was to investigate the existence of a region of effect in the pedicle screw pullout procedure and the sensitivity of this region to the material properties of the cancellous bone. Accordingly, an experimentally validated three-dimensional FE model of a screw-bone unit was established and used to determine the RoE in screw pullout simulations.

## Methods

### Experimental study

**Figure 1:**
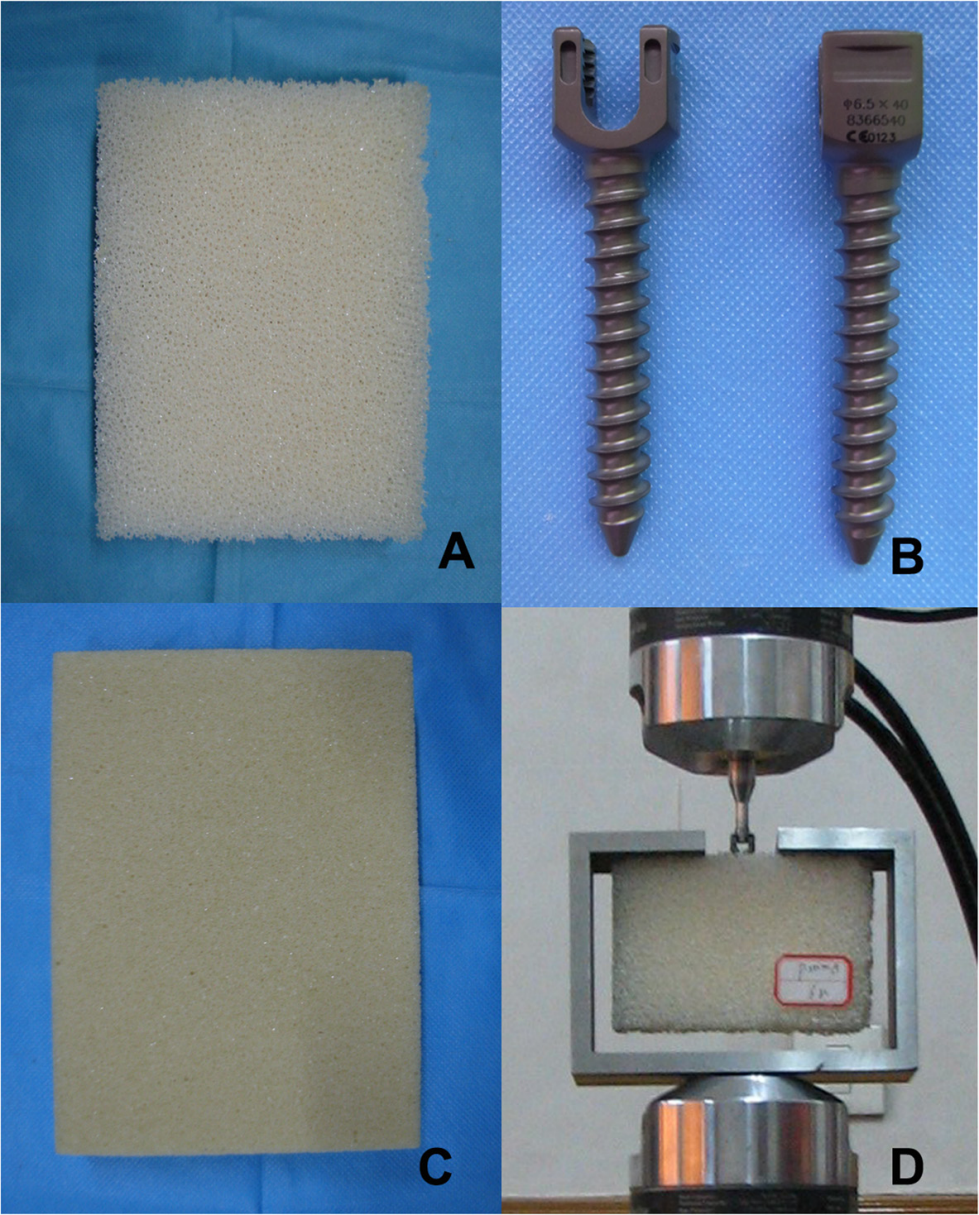
**The pullout experimental setup configuration. A**: Foam with lower density; **B**: CDH Ø 6.5 × 40 mm pedicle screw; **C**: Foam with higher density; **D**: Pullout test configuration.

Sixteen conventional pedicle screws (CDH Ø 6.5 × 40 mm) and polyurethane foam (an alternative test medium, analogous to human cancellous bone, with uniform and consistent mechanical properties (according to ASTM F-1839)) were used. Two different types of polyurethane foam with different properties — ρ = 0.16 g/cc, porous cell size 0.5 to 2.0 mm, and Young’s modulus of 23 MPa; and ρ = 0.32 g/cc, porous cell size 0.5 to 1.0 mm, and Young’s modulus of 137.5 MPa, [20,21], which approximated to human osteoporotic cancellous bone characteristics and normal cancellous bone characteristics, respectively, were utilized (Figure 1). In accordance to the ASTM-F543 standard testing procedure, the pullout force was obtained of the pedicle screws from these two different densities of solid rigid polyurethane foam blocks (10 pfc, Sawbones Worldwide, Pacific Research Laboratories Inc).

The screw-foam samples were prepared according to the surgical procedure. First, guiding holes with diameters of 3.5 mm and depths of 50 mm were drilled into the polyurethane blocks, and these guiding holes were then tapped manually, using the taps provided by the manufacturer (CD Horizon legacy MD-8 system, Medtronic Sofamor Danek Inc., Memphis, TN, USA). Finally, the 6.5 × 40-mm pedicle screws with 13 threads were screwed into the the polyurethane block to the full dept. The guiding holes were 10 mm deeper than the desired screw insertion depth, to ensure that the tip of the screw was not pressed against the bottom of the guiding hole, thus avoiding the generation of undesired pretension on the foams block [22]. Sixteen pedicle screws were randomly divided into two groups and were screwed into the polyurethane blocks.

**Figure 2:**
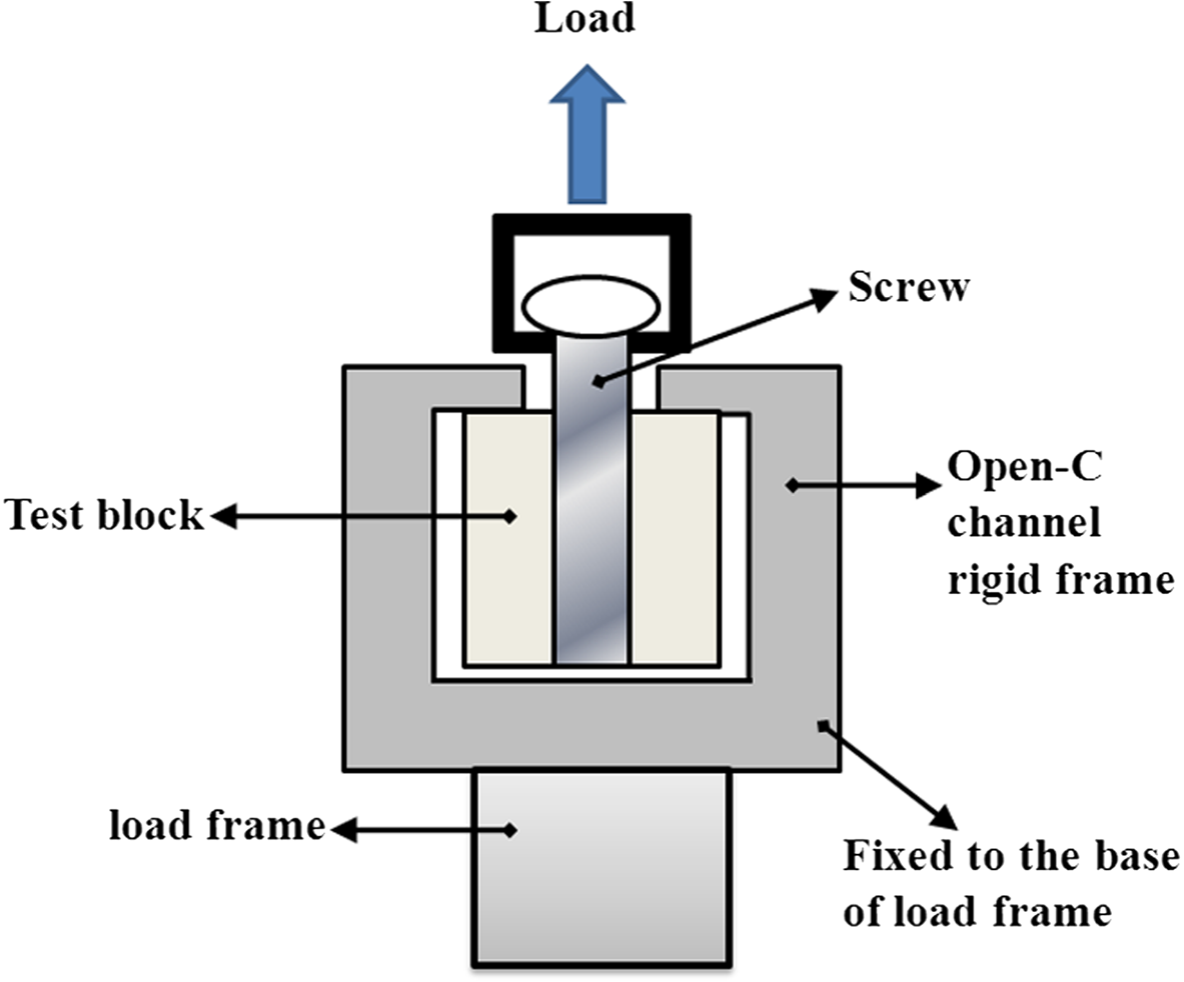
Schematic of test configuration.

Figure 2 shows the pullout experimental setup configuration. The pullout test jig consisted of an open C-channel rigid frame secured to the base of loading frame and a special fabricated rod, with one end threaded to screw into the head of the pedicle screw of the block sample and its enlarged end rigidly clamped to the upper moving cross head of the loading frame (MTS MiniBionix 858, MTS systems Corp, Eden Prairie, MN, USA). The upper moving cross-head was controlled to displace in the pull-out direction at a constant rate of 0.01 mm/s [22], and the corresponding reaction force was documented at a sampling rate of 10 Hz. Load–displacement graphs were plotted for all the samples, and the average maximum pullout force was computed for the two different screw-foam samples.

### FE model design

Because the screw-bone interface was periodically symmetric, and a linear correlation existed between the number of threads and pullout strength [15,16], a quarter unit model with only one thread was established for computational efficiency. Three FE models were developed: two screw-foam models were used for the validation study, and one screw-bone model was used for the material sensitivity analysis. Using Pro/Engineer software (PTC, Needham, MA, USA), based on the dimensions and profiles of the screw thread specifications of the standard pedicle screws [16,23], the correct geometries of the pedicle screws were created. Separately, a quarter cylindrical block, 18 mm in diameter and 4 mm in length, was created. A threaded hole that matched the pedicle screw profile was created at the center of the block. The created three-dimensional geometries were then imported into ANSYS/LS-Dyna (ANSYS Inc., Canonsburg, PA, USA) for FE mesh generation.

**Figure 3:**
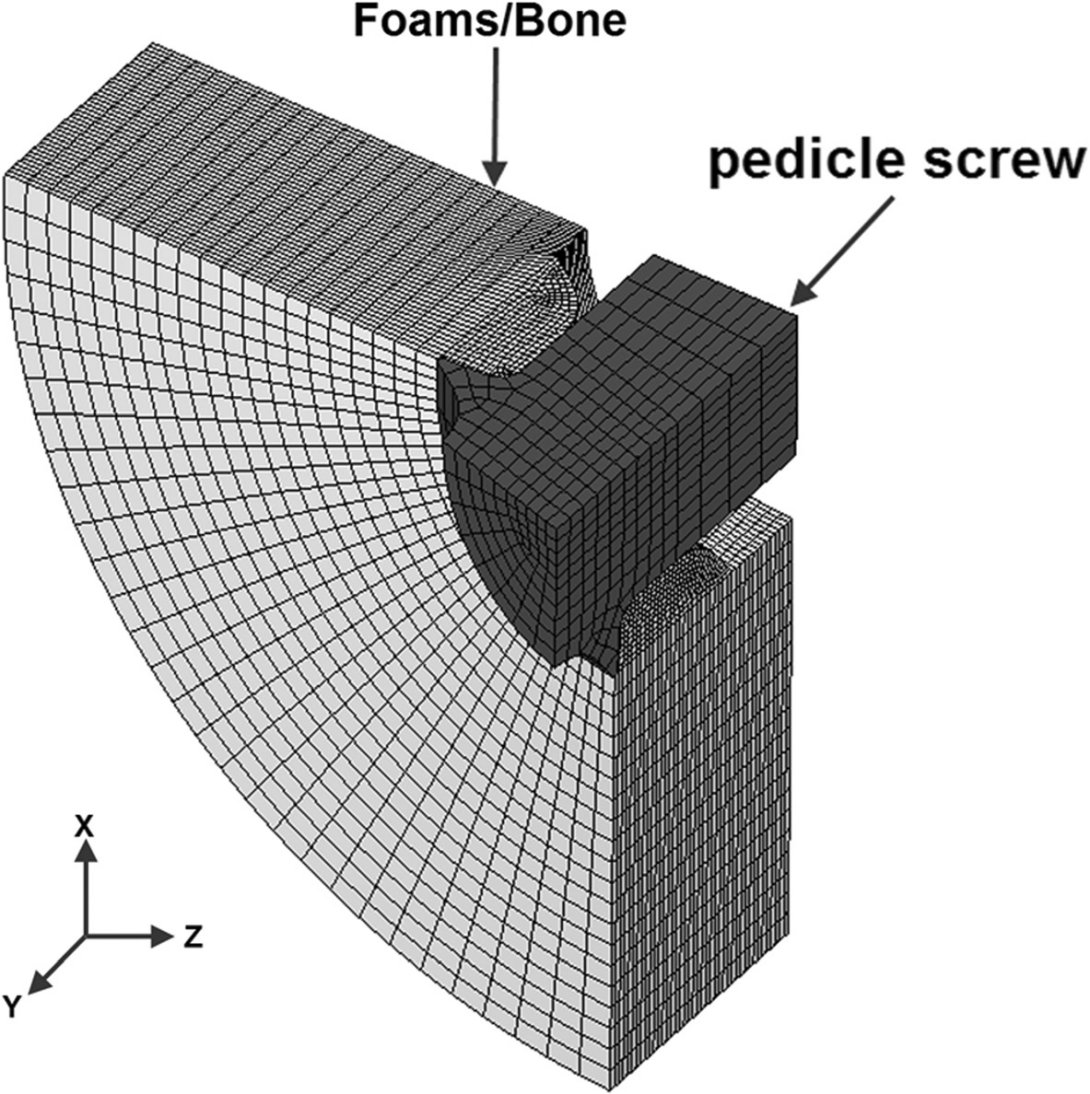
Finite element model of screw-foams/bone.

For a reasonable representation of the actual matching geometry between the foam/bone and screw threads, a fine mesh (0.1 mm in average length) was adopted, while a relatively coarser mesh (0.3 mm in average length) was adopted for the other regions. For the region between the interfaced threads, significant mesh refinement was used to obtain better stress distribution around the cancellous bone tissue in this area [24,25]. The FE meshed screw and block models were assembled to generate the final FE screw-foam/bone model. In this study, an eight-noded isoparameteric brick solid element was chosen. Convergence testing was performed, and the final FE model consisting of 99,354 elements and 36,506 nodes (Figure 3), was subsequently used for further simulation study.

To simulate the contacts between the foam/bone and screws at their interfaces under pullout loading conditions, the surface-to-surface contact relationship was employed in the model. Contact pairs were defined between the foam/bone and screws, with the trailing edge of the screw chosen as the master surface and those elements of the foam/bone chosen as the slave surface.

The material properties of the foams adopted from the literature [22], which were used for the FE screw-foam model, are shown in Table 1.

**Table 1:** Material properties of Ti alloy screw and Foams used in the current simulation

**Material properties**	**Density (g/cm**^**3**^**)**	**ν**	**E (MPa)**	**σ y (MPa)**	**Reference**
Ti alloy screw	4430	0.3	110000	860	Chatzitergos et al. [16]
Foam LD	0.16	0.2	57	2.2	Chapman et al. [23]
Foam HD	0.32	0.2	267	5.9	Chapman et al. [23]

### FE model validation

**Figure 4:**
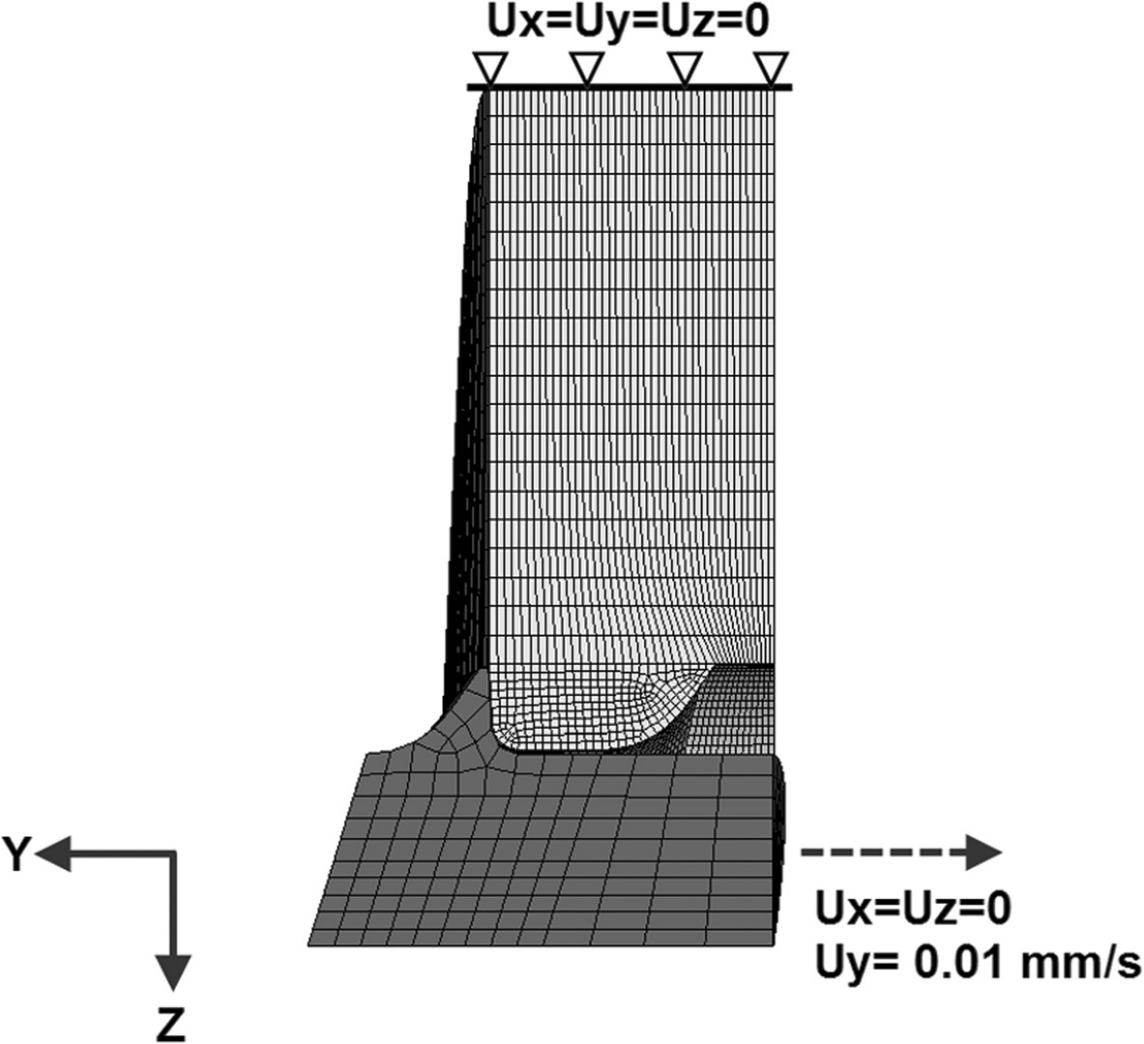
Boundary conditions and loading of the pullout simulation.

The validation of the FE screw-foam model was conducted by evaluating the predicted screw pullout strengths and the pullout force-displacement curves against the experimental data obtained from the current test and from Hashemi et al.’s study [22]. By applying similar boundary and loading conditions for the FE screw-foam model as in the experiment test, as shown in Figure 4. The global XYZ coordinate system was set with the *y*-axis acting along the axis of the screw length, and the *x*- and *z*-axes pointing radially. The nodes in the circumferential surfaces of the foam were fixed in all degrees of freedom. The degrees of freedom in *x-* and *z-* direction translation were restricted, and a constant velocity of 0.01 mm/s, with displacement of 2.7 mm along the positive *y-*direction translation was prescribed to the pedicle screw [22].

The maximum reaction force, defined as F_Q_, was extracted from all of the fully restrained nodes in the circumferential surfaces during the screw pullout procedure. The pullout force for a complete screw-foam model with N threads inserted into the cancellous bone, defined as F, was calculated based on equation (1):

(1) $$F=4\times F_{Q}\times N$$

with N = 13 to correlate with those in the current experiment and in the studies by Hashemi et al. [22].

### Calculation of RoE in cancellous bone: material sensitivity analysis

For the material sensitivity study, the foam material properties of the validated screw-foam model were changed appropriately to emulate those of the cancellous bone. In this study, the cancellous bone was assumed to be homogeneous and, isotropic with an elastic-perfect plastic material, similar to those described in Hayes et al.’s study [26]. The material properties of cancellous bone, including its apparent density, Young’s modulus and yield strength, as derived from the literature [20,21], were adopted. Young’s modulus of cancellous bone ranged from 60 MPa to 260 MPa with an increment of 40 MPa to simulate the 3 age groups [27,28] in this study. A failure strain of 60% was assigned to the cancellous bone to define the failure behavior during the simulation [29]. The material properties of titanium alloy were used to model the screw. A friction coefficient of 0.2 was assigned for the contact surface between the screw and bone [30]. The material properties of all of the components described in the screw-bone model are provided in Table 1 and Table 2. Similar boundary and loading conditions were applied to the screw-bone model to investigate the stress distribution in the bone tissue during pullout simulations in models with different material properties (Figure 4). During the simulation, the pullout forces and the stress distribution were documented along the radial path around the pedicle screw in cancellous bone.

**Table 2:** Material properties of human cancellous bone used in the current simulation

**Age**	**Density (g/cm**^**3**^**)**	**ν**	**E (MPa)**	**σ y (MPa)**	**Reference**
Age >75 yr	0.06	0.2	60	0.615	Morgan et al. [20]
	0.08		100	1.015	
Age 50–75 yr	0.10	0.2	140	1.415	Hou et al. [21]
	0.12		180	1.815	
Age <50 yr	0.14	0.2	220	2.215	
	0.16		260	2.615	

During the screw pullout procedure, the reaction force at the screw head gradually increased to the maximum and then decreased, and the pullout force was defined as the maximum reaction force. The region of effect (RoE) was defined by the region surrounding the cancellous bone tissue with a von Mises stress value > 0.01 MPa at the maximum reaction force. Then, based on the surrounding region, the RoE was estimated to be a circular area with a radius of *Δr* beyond the outer pedicle screw radius, and the volume of the RoE for the complete screw, with a purchase length of L, defined as V_A_, was calculated from equation (2):

(2) $$V_{A}=\left[\pi {\left(r+\mathrm{\Delta r}\right)}^{2}-\pi r^{2}\right]\times L$$

Where:

*r*: Outer radius (mm) of pedicle screw;

*Δr*: Radius (mm) of RoE;

*L*: The purchase depth (mm) of pedicle screw into the cancellous bone of vertebral body

At maximum pullout force, the stress distribution and magnitude in the cancellous bone along *Δr* beyond the outer perimeter pedicle screw was also documented.

## Results

### Experimental study and FE model validation

**Figure 5:**
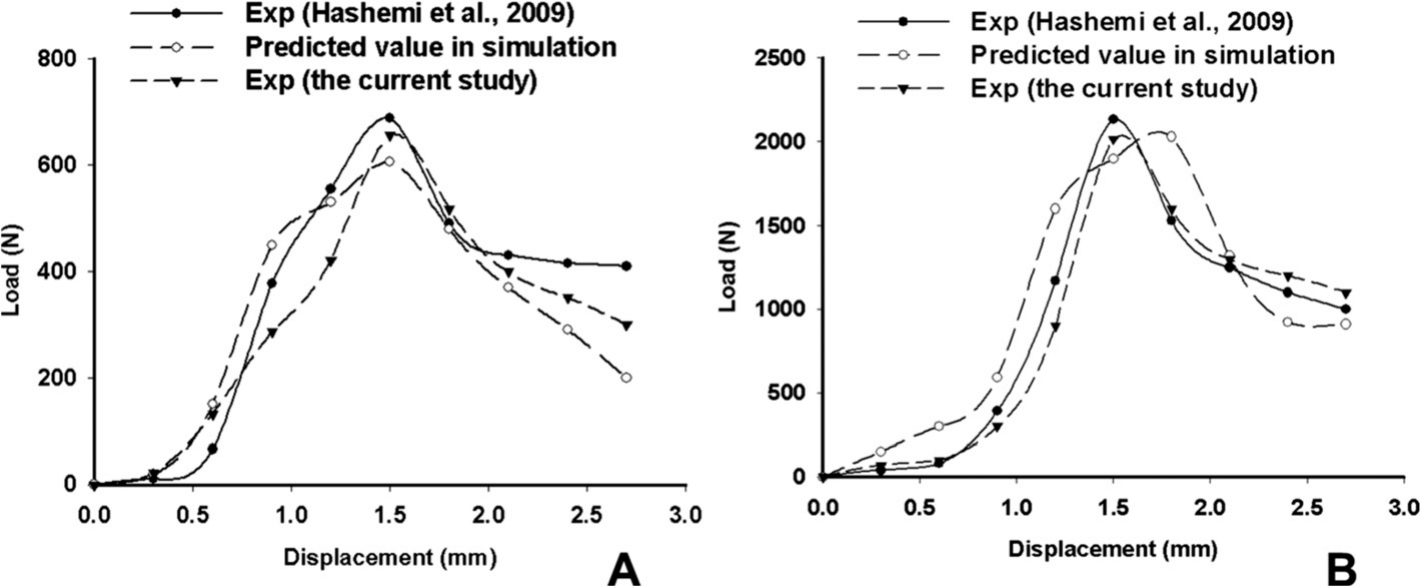
**The load–displacement curve of pullout tests and simulation. A**: in the lower density group; **B**: in the higher density group.

The comparisons between the current FE predicted and experimental screw pullout strengths and the load–displacement graphs against those obtained from Hashemi et al.’s study [22], are shown in Table 3 and Figure 5. All of the load–displacement graphs showed similar trends, with the same orders of magnitude in the loads and reaching maximum values for displacement in the region of 1.5 to 2 mm. The experimental measured pullout forces for the CDH 6.5 screws were 2,015 ± 95.7 N and 657 ± 69.4 N in high-density foam and low-density foam, respectively. Correspondingly, the FE predicted pullout forces were 2,028.8 N and 607 N, respectively.

**Table 3:** Comparison of predicted screw pullout strength against those in current test and the published literature

	**Validation against our test**		**Validation against the test in literature**	
	**The current experiment (n = 8)**	**Predicted value**	**Hashemi et al.’s experiment (n > 4)**	**Predicted value**
High density	2015 ± 95.7 N	2028.8 N	2132.5 ± 119.3 N	2028.8 N
Low density	657 ± 69.4 N	607 N	688.2 ± 91.4 N	607 N

### The stress distribution around the pedicle screw and material sensitivity analysis

The predicted pullout forces from the FE screw-bone models of different bone material properties are shown in Table 4. The results showed increased pullout force with the Young’s modulus (the stiffness) of the bone block. The pullout forces with the bone’s Young’s modulus range from 220 to 260 MPa (simulating age groups < 50 years old) were 702 N and 1040 N, respectively. These values are 2 to 3 times greater than those in the simulated 75 years old > age > 50 years old group (442 N and 520 N) and are 3 to 4 times greater than those in the simulated group of age > 75 years old (221 N and 273 N).

**Table 4:** The pullout force (PF) in blocks with different material properties

	**Age > 75 yrs**		**75 yrs > age > 50 yrs**		**50 yrs > age**	
E (MPa)	60	100	140	180	220	260
PF (N)	221	273	442	520	702	1040

**Figure 6:**
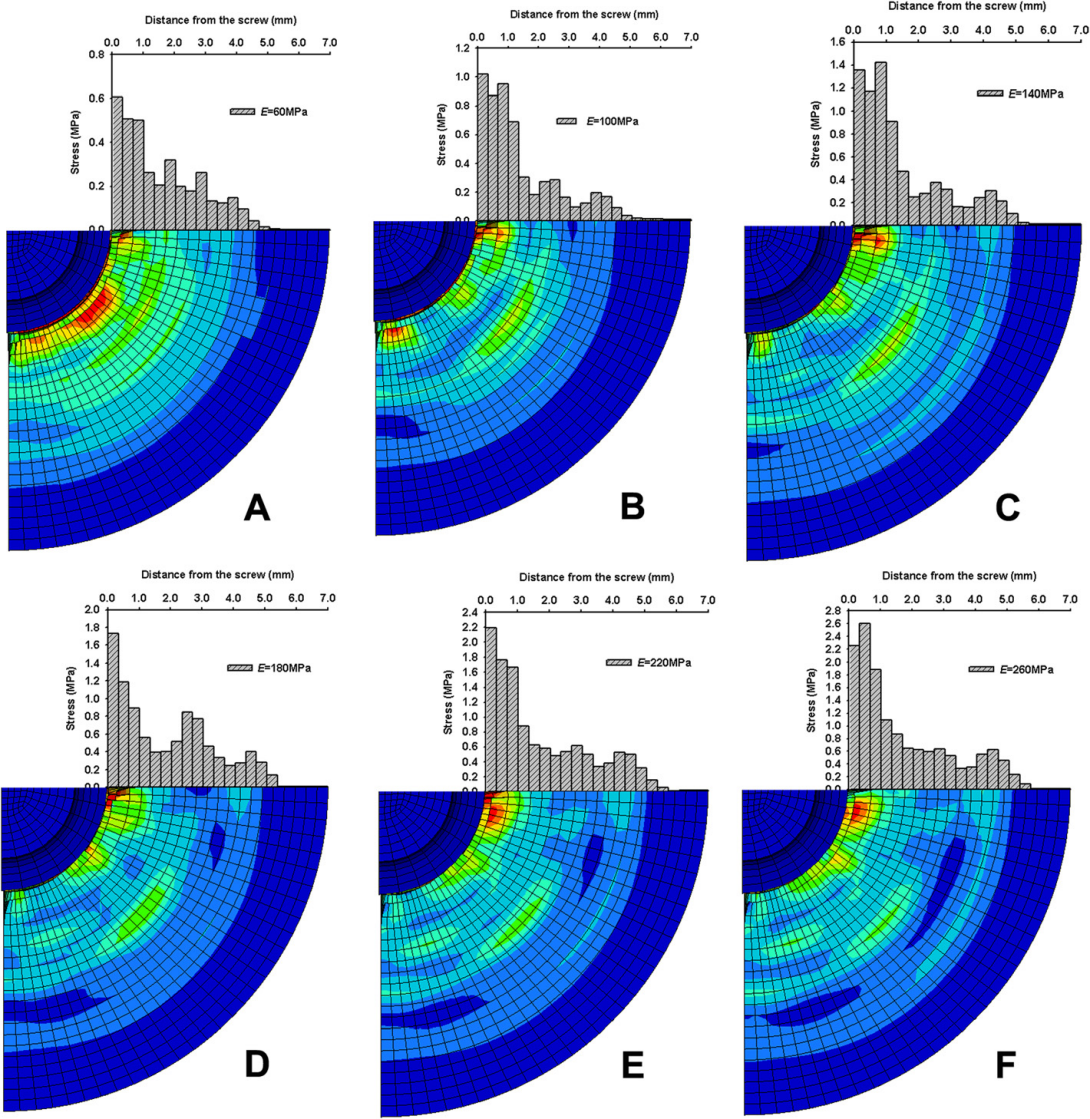
**Stress distribution and magnitude in the cancellous bone during pullout procedure. A**: E = 60 MPa; **B**: E = 100 MPa; **C**: E = 140 MPa; **D**: E = 180 MPa; **E**: E = 220 MPa; **F**: E = 260 MPa.

Figure 6 shows the stress distribution around the pedicle screw for all of the simulated FE screw-bone models, demonstrating that the magnitude of the von Mises stress distribution in cancellous bone decreased gradually with similar patterns in the radial direction for all of the simulated FE screw-bone models. The highest magnitude of stress in the cancellous bone tissue was found in the region nearest the pedicle screw and it decreased as the distance between the bone tissue and the screw increased.

The results show that there existed a hypothetical effective region with radius of RoE (*Δr*) and volume of RoE (*V*A) for the stabilization of the pedicle screw in screw-bone fixation. Table 5 shows that the predicted *Δr* and *V*A in cancellous bone around the pedicle screws increased with the stiffness of the bone block. *Δrs* was 4.73, 5.06 and 5.4 mm in the simulated age group > 75 years old, 75 years old > age > 50 years old and age < 50 years old, respectively. The corresponding *V*A was 6.67, 7.35 and 8.07 ml, respectively.

**Table 5:** The RoE in blocks around the pedicle screws with different material properties

	**Age > 75 yrs**		**75 yrs > age > 50 yrs**		**50 yrs > age**	
E (MPa)	60	100	140	180	220	260
*Δr* (mm)	4.73	4.73	5.06	5.06	5.40	5.40
*V*A(ml)	6.67	6.67	7.35	7.35	8.07	8.07

## Discussion

In the current study, an experimentally validated FE screw-bone model was established for the pullout simulation study, and the region of effect was investigated for pedicle screw stabilization around the pedicle screw during a pullout procedure. The results also showed that there existed a circular region in the cancellous bone around the pedicle screw, and this circular region was sensitive to the material properties of the cancellous bone.

The current study could provide spine surgeons with a clinical reference for pedicle screw instrumentation and augmentation. Many experimental studies have demonstrated that the stiffness and strength of pedicle screw-bone fixation could be significantly enhanced for screws augmented with various cements. Recently, several clinical studies have been conducted to investigate the application of cement augmentation for pedicle screw techniques [2,9,31]. However, cement has not been widely applied for pedicle screw augmentation clinically because of safety considerations. Moreover, a practical and reliable surgical technique for the augmentation of pedicle screws with cement has not yet been developed. Cement has many advantages for pedicle screw augmentation due to its high strength and rapid solidification, but an excessive volume of cement injection and posterior leakage of cement into the spinal canal can be catastrophic [32,33]. Based on the results of the current study, the augmentation of the hypothetical region of effect could improve the stability of pedicle screws, and an appropriate volume of cement injection could be established for clinical reference.

The FE screw-foam models, based on pedicle screw CHH 6.5, were developed for pullout simulations in the current study and were validated against the experimental pullout test conducted in Hashemi et al.’s study [22]. The screw pullout forces, extracted from two density screw-foam experimental tests and from simulated FE screw-foam models, showed direct proportionality to the strength of the material properties of the foam. The predicted pullout force and the load–displacement curves from the FE screw-foam models were of the same order of magnitude and showed similar trends with experimental results in Hashemi et al.’s study [22], as shown in Figure 5. These results demonstrated that FE screw-foam models were applicable for screw-bone pullout simulation, with appropriate material properties, allowing for the assumption of modeling the microstructure of cancellous bone as a cellular solid [34].

By changing the material of the mesh for the foam to that of cancellous bone, a modified FE screw-bone model was adopted for the pullout simulation to predict the pullout force and the hypothetical effective region (RoE) for screw stabilization. The FE computed results showed that the stress distribution pattern in cancellous bone around the pedicle screw was circular in shape. The stress magnitude was the highest in the region near the pedicle screw, and it decreased with the distance from the screw. This result supported the hypothesis that there existed a circular region around the pedicle screw, called the region of effect, which could play a pivotal role in screw stabilization.

As shown in the results, the range of RoE and screw pullout forces were affected by the mechanical properties of cancellous bone. However, Morgan et al. [20] and Hou et al. [21] reported that the material properties (density, stiffness, ultimate strength and yield strength) of cancellous bone changed with age. Therefore, with the different values of cancellous bone, Young’s modulus (in various appropriate age groups) was used in the current study, and the corresponding predicted stress magnitudes in the cancellous bone showed that the RoE decreased with bone properties. The present study showed that ***Δr*** ranged from 4.73 to 5.40 mm for standard 6.5 lumbar of specified thread profiles, and this range could vary depending on the screw type and thread profile (such as the thread shape factor and the inclination of the leading edge), which will be investigated in a further study.

In this study, the predicted RoE is based on the stress contour during the pullout simulation. The region with higher stress (near the pedicle screw) plays an important role in stabilizing the pedicle screw. The region with lower stress (distance away from the pedicle screw) carries less of a load in stabilizing the pedicle screw. We adopted the region with a von Mises stress value < 0.01 MPa to be ineffective in the stabilization of the pedicle screw. Based on the material properties of the cancellous bone, with minimum yield stress of the cancellous bone in the age > 75 years old group of 0.615 MPa, and the value of 0.01 MPa less than 2% of the yield stress, the region with von Mises stress value < 0.01 MPa could therefore play a minor role in pedicle screw stabilization.

The development of any computational model requires a number of assumptions regarding the geometry, materials, and interactions between components. In the present study, the model was a quarter screw-foam/bone unit excluding the cortical bone. The commonly used standard of the 6.5 mm pedicle screw type in clinical application for fixation in osteoporotic patients was used in the modeling. Elastic-perfect plastic material properties were adopted for cancellous bone tissue in the current study, based on the Hayes et al.’s study [26], and these properties were sufficient for the relatively simple loading conditions for screw pullout. In the present simulation, 60% strain was used as the failure standard of the cancellous bone tissue [29]. Although the yield stress in cancellous bone occurs at strains of 5-10%, the strain usually exceeds 60% before failure in the cellular structure of cancellous bone [26,35]. The friction coefficient between the screw and cancellous bone was set at 0.2, based on previous studies [15,36]. In addition, a quarter screw-bone unit with fine mesh (mesh size: 0.1 mm; elements number: 99,354) was established in the present study for computational efficiency. In the present study, the RoE was calculated without considering the material properties of the injection cement. Although the calculated RoE in the current simulation was a circular region around the outer radius of the pedicle screw, the RoE could change with the injection of various cements. We speculate that the properties of injection could influence the RoE, that cement with a higher Young’s modulus, like PMMA, could enlarge the RoE in the cancellous bone, and that the RoE of biodegradable cement, such as CPC or hydroxyapatite, could vary with absorption of the biomaterials. Cancellous bone with various cement augmentations for pedicle screws requires further investigation.

## Conclusions

In conclusion, the RoE was calculated based on an experimentally validated FE model in the present study. The results showed that there existed a circular region of effect around the pedicle screw during the pullout simulation. The RoE was sensitive to the material properties of the cancellous bone. The results suggested that the RoE could play a significantly role in pedicle screw augmentation, and the proper amount of injection cement for augmentation could be estimated in the treatment of osteoporosis patients for spine surgery.

## Competing interests

The authors declare that they have no competing interests.

## Authors’ contributions

SL, WQ and YZ have made substantial contributions to conception and design, and acquisition of data, and analysis and interpretation of data; ZXW have been involved in drafting the manuscript and revising it critically for important intellectual content; YBY have given final approval of the version to be published; and WL agree to be accountable for all aspects of the work in ensuring that questions related to the accuracy or integrity of any part of the work are appropriately investigated and resolved. All authors read and approved the final manuscript.

## Authors’ information

SL: Department of Orthopaedics, Xijing Hospital, Fourth Military Medical University, Xi’an, China;

WQ: Surgery Department of 520th Hospital of PLA, Mian yang, China;

YZ: Department of Orthopaedics, Xijing Hospital, Fourth Military Medical University, Xi’an, China;

ZXW: Department of Orthopaedics, Xijing Hospital, Fourth Military Medical University, Xi’an, China;

YBY: Department of Orthopaedics, Xijing Hospital, Fourth Military Medical University, Xi’an, China;

WL: Department of Orthopaedics, Xijing Hospital, Fourth Military Medical University, Xi’an, China.

Shuai Liu, Wei Qi and Yang Zhang are the co-first authors.
